# ICF, An Immunodeficiency Syndrome: DNA Methyltransferase 3B Involvement, Chromosome Anomalies, and Gene Dysregulation

**DOI:** 10.1080/08916930802024202

**Published:** 2008-04-23

**Authors:** Melanie Ehrlich, Cecilia Sanchez, Chunbo Shao, Rie Nishiyama, John Kehrl, Rork Kuick, Takeo Kubota, Samir M. Hanash

**Affiliations:** 1 Hayward Human Genetics Program, Tulane Medical School, New Orleans, LA 70112, USA; 2 Center for Gene Therapy, Tulane Medical School, New Orleans, LA 70112 USA; 3 RIKEN, Plant Science Center, Ibaraki 305-0074, Japan; 4 National Institutes of Allergy and Infectious Diseases, Bethesda, MD 20892, USA; 5 Biostatistics Core, University of Michigan Comprehensive Cancer Center, University of Michigan, Ann Arbor, MI 48109, USA; ^6^Epigenetic Medicine, University of Yamanashi, Yamanashi, Japan; ^7^Molecular Diagnostics, Fred Hutchinson Cancer Research Center, Seattle, WA 98109, USA

**Keywords:** Immunodeficiency, constitutive heterochromatin, cancer, DNA methyltransferases, chromosomal rearrangements, DNA demethylation

## Abstract

The immunodeficiency, centromeric region instability, and facial anomalies syndrome (ICF) is the only disease known to result from a mutated DNA methyltransferase gene, namely, *DNMT3B*. Characteristic of this recessive disease are decreases in serum immunoglobulins despite the presence of B cells and, in the juxtacentromeric heterochromatin of chromosomes 1 and 16, chromatin decondensation, distinctive rearrangements, and satellite DNA hypomethylation. Although DNMT3B is involved in specific associations with histone deacetylases, HP1, other DNMTs, chromatin remodelling proteins, condensin, and other nuclear proteins, it is probably the partial loss of catalytic activity that is responsible for the disease. In microarray experiments and real-time RT-PCR assays, we observed significant differences in RNA levels from ICF vs. control lymphoblasts for pro- and anti-apoptotic genes (*BCL2L10*, *CASP1*, and *PTPN13*); nitrous oxide, carbon monoxide, NF-κB, and TNFa signalling pathway genes (*PRKCH*, *GUCY1A3*, *GUCY1B3*, *MAPK13*; *HMOX1*, and *MAP4K4*); and transcription control genes (*NR2F2* and *SMARCA2*). This gene dysregulation could contribute to the immunodeficiency and other symptoms of ICF and might result from the limited losses of DNA methylation although ICF-related promoter hypomethylation was not observed for six of the above examined genes. We propose that hypomethylation of satellite 2at1qh and 16qh might provoke this dysregulation gene expression by *trans* effects from altered sequestration of transcription factors, changes in nuclear architecture, or expression of noncoding RNAs.

## ICF: Introduction to the syndrome

The immunodeficiency, centromeric region in stability and facial anomalies syndrome (ICF) is a rare recessive disease characterized by targeted chromosome breakage [1]. The majority of cases of ICF that have been studied involve mutations in one of the three main DNA methyltransferase genes, *DNMT3B* [2–4]. These *DNMT3B* mutations are usually missense changes within the coding portion of the gene [5–8]. This is the only known genetic disease in humans involving mutations in one of the genes encoding an enzyme that methylates cytosine residues in DNA.

**Figure 1:**
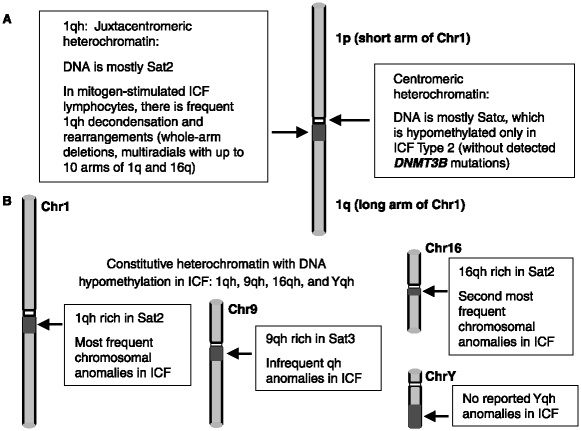
Hypomethylated DNA in constitutive heterochromatin in ICF. Cartoon illustrating the constitutive heterochromatin regions that display ICF-specific hypomethylation and chromosome abnormalities. Dark gray box, juxtacentromeric (pericentromeric) heterochromatin; white box, centromere.

ICF is an immunodeficiency disease that has been described in fewer than 50 patients world-wide [9, 10] since it was first described in the late 1970s [11, 12]. It is diagnosed by immunoglobulin deficiency that is usually seen in the presence of normal B- and T-cell counts, characteristic chromosomal abnormalities in the vicinity of the centromere of certain chromosomes, and, usually, also facial anomalies. The immunodeficiency of ICF patients is largely responsible for their frequent mortality in early childhood. The chromosomal abnormalities are instability that is almost exclusively found in the juxtacentromeric heterochromatin (qh) regions of chromosomes 1 and 16, and sometimes 9 (Figure 1). In addition, all studied ICF tissues and cell cultures display hypomethylation of satellite 2 DNA (Sat2) in 1qh and 16qh, the related satellite 3DNA (Sat3) in 9qh, and, formales, in Yqh satellite DNA [13, 14].

In this review, we will briefly describe the ICF phenotype, the nature of known ICF-associated mutations in *DNMT3B*, and why it is likely that ICF is actually due to loss of the enzymatic activity of DNMT3B and not to alterations in its specific protein–protein interactions. The relationships of DNA hypomethylation and chromosome abnormalities in the ICF syndrome and in cancer will also be discussed. Lastly, the question of abnormal gene expression in ICF lymphoblastoid cells will be addressed in some detail with previously unreported data from an expression microarray analysis and an inferred model of how ICF-related DNA hypomethylation leads to the disease.

## ICF-linked DNMT3B mutations

### DNMT3B overview

ICF patients with *DNMT3B* mutations [2, 4, 10] are sometimes referred to as exhibiting ICF type 1 disease [7]. These patients are usually compound heterozygotes with various mutations within the gene [5, 6, 10]. In mice, *Dnmt3b* is an essential gene for normal development [15]. Insertional in activation of *Dnmt3b* or *Dnmt1* results in prenatal death soon after implantation [15]. In murine knock-outs of the third major DNMT gene, *Dnmt3a*, death is observed several weeks after birth. In humans, if ICF-causing mutations in *DNMT3B* did not leave residual activity, embryonic lethality would probably result. This residual DNA methylation activity has been observed *in vitro* [16] and is consistent with results from *in vivo* mouse models [17]. Therefore, we predict that homozygous null *DNMT3B* mutations would lead to spontaneous abortions.

Human DNMT3B and murine Dnmt3b (94% identity) and human DNMT3A and murine Dnmt3a (98% identity [18]) have predominant roles in *de novo* methylation of DNA (methylation of CpG dyads that were symmetrically unmethylated) [19]. These are enzymes are especially important during embryogenesis and gametogenesis [20, 21] although their activity is not limited to these stages in development. *DNMT3B* and *DNMT3A* are not redundant in terms of function [22], as validated by the finding that *DNMT3B* mutations suffice to cause ICF. They differ in expression patterns during murine development [23] although they can interact and stimulate each other's activity [24]. They have some distinct preferences for sequences flanking the CpG dinucleotide and for chromosomal regions [19, 20, 25]. They also differ in relative activity *in vitro* toward DNA substrates in nucleosomes vs. naked DNA [26]. Complicating analysis of the function of *DNMT3B*/*Dnmt3b* and *DNMT3A*/*Dnmt3* a gene products are the numerous isoforms that they encode, which show non-coordinate expression [22]. For example, one of the DNMT3B isoforms is missing conserved motifs in the catalytic domain but evidence suggests that it is still biologically important [27].

### DNMT3B mutations in ICF patients (ICF type 1)

ICF type 1 is the only form of ICF whose genetic etiology is known. It involves biallelic *DNMT3B* mutations [10]. Unless otherwise noted, ICF will denote type 1 in this review. The ICF-linked *DNMT3B* mutations are often missense mutations and are usually found in the part of the gene encoding the catalytically active C-terminal portion of the protein, namely, one of ten motifs conserved among all cytosine-C5 methyltransferases [1, 2, 4, 5, 10, 15, 28].

The involvement of DNA hypomethylation in the phenotype of ICF is supported at the cytogenetic level. ICF-specific rearrangements in mitogen-treated lymphocytes from patients are the same in frequency, spectrum and chromosomal specificity as those that we found in a normal pro-B lymphoblastoid cell line treated with the DNA methylation inhibitors 5-azacytidine or 5-azadeoxycytidine [29, 30]. The invariant hypomethylation of certain portions of the genome in ICF cells and tissues, most notably Sat2 [1, 13], is also consistent with ICF being due to DNA methylation deficiency.

### Noncatalytic functions of DNMT3B

DNMT3B has repressor activity that is independent of its DNA methyltransferase activity [31]. Accordingly, DNMT3B has many specific protein-interaction domains, which are usually outside the C-terminal catalytic domain [32–34]. The specific binding partners of DNMT3B include the other DNA methyltransferases DNMT1 and DNMT3A, histone deacetylases HDAC1 and HDAC2, HP1α, the chromatin remodelling protein hSNF2H, the condensing complex hCAP-C/hCAP-E, and the mitotic chromosome-associated KIF4A [33–35]. DNMT3B is subject to sumoylation [32], which involves addition of a small SUMO protein to alysine residue in a particular protein motif [36]. Sumoylation can affect protein–protein interactions, protein activity and protein localization [37]. The sumoylation and PWWP chromatin targeting domains in the N-terminal half of DNMT3B probably are responsible for intra-nuclear and heterochromatin targeting of the enzyme, respectively [32, 38, 39], which can occur in a cell type-specific manner [31]. Satellite DNA methylation does not appear to be necessary for targeting of Dnmt3b to heterochromatin in murine embryonal stem cells [31] but Sat2 hypomethylation might be involved in the exaggerated targeting of 1qh and 16qhto intranuclear bodies [40], as will be described below.

The only ICF-associated missense mutation outside the catalytic C-terminal half of DNMT3B (S282P) is within the PWWP domain and was found in both DNMT3B alleles in two related ICF patients [28]. The analogous mutation in mouse cells resulted in the loss of detectable targeting to constitutive heterochromatin in interphase and meta-phase [38]. This redistribution could explain the hypomethylation of Sat2 in the juxtacentromeric constitutive heterochromatin of these two ICF patients [28] despite the persistence of methyltransferase activity *in vitro* [38].

The functional importance of the non-catalytic roles of DNMT3B/Dnmt3b was illustrated by studies of differentiation of rat pheochromocytoma cells (PC12) into neuronal cells. Induction of differentiation in PC12 cells mediated by nerve growth factor was inhibited by antisense or small interfering RNA (siRNA) for Dnmt3b [41]. This inhibition could be largely reversed by transfection with a plasmid encoding Dnmt3b, either wild-type or mutated in the C-terminal catalytic domain so as to inactivate catalytic activity, but not by mutants missing the central ATRX domain or N-terminal PWWP domain. One of the non-catalytic, neuronal differentiation-related targets of the Dnmt3b is the T-cadherin promoter whose activity was repressed by Dnmt3b irrespective of this promoter's methylation status [42].

Generally, mutant DNMT3B proteins from ICF cells are still able to engage in normal protein–protein interactions [34]. One exception is an ICF mutation that altersthe amino-terminal region of DNMT3B's catalytic domain, and, in amouse mutant interferes with acatalysis-enhancing interaction of Dnmt3a and Dnmt3b [17]. The other exceptions are two mutations in the C terminal half of DNMT3B that did not appreciably reduce catalytic activity but did greatly reduce its interaction with Dnmt3L [43] (see below). These exceptions further implicateloss of DNA methylation, and not some other biological activity of DNMT3B, as the upstream molecular defect causing the ICF syndrome.

### Stimulation of DNMT3B catalytic activity by interaction with DNMT3L

There is a specific DNMT3L interaction domain located in the C-terminus of Dnmt3b/DNMT3B [44]. DNMT3L is anon-catalytic protein needed for establishment of DNA methylation patterns in the germline, including maternal methylation imprinting and normal levels of methylation of satellite DNA sequences [20]. DNMT3L stimulates the catalytic activity of Dnmt3a and Dnmt3b methyltransferases [45]. This may involve contact of DNMT3B and DNMT3L leading to a changes in the higher-order organization of DNMT3B, just as DNTM3L reorganizes the oligomerization of the DNMT3A2 isoform of DNMT3A [46]. Importantly, two ICF-associated *DNMT3B* mutations (A766P and R840Q) that result in proteins with approximately wild-type basal methylation activity exhibit decreased association with DNMT3L and a strong decrease in stimulation by DNMT3L [43]. These results implicate interaction of DNMT3B with DNMT3L as critical for normal DNA methylation that is protective against the ICF phenotype.

### ICF type 2: Not associated with DNMT3B mutations

About 40% of ICF patients have no mutations in exons of *DNMT3B* [7, 10]. There might be mutations in the promoter or other transcription control elements or mutations affecting splicing or polyadenylation. However, for several of these ICF patients without detected *DNMT3B* mutations, the most common isoform of DNMT3B RNA was still observed [7], and for the one patient examined in two putative promoter regions of *DNMT3B*, no mutations were found [8]. Moreover, mRNA froman ICF patient without detected mutations in *DNMT3B* was examined by RT-PCR with primers or amplicon sizes specific for several of the DNMT3B RNA splice variants 3B4, 3B3, and3B1 [47]. No evidence for pathogenic alterations in DNMT3B splicing patterns was uncovered.

ICF patients without *DNMT3B* coding region mutations seem to be derived from a different subtype of the disease. Lymphocytes or fibroblasts from all nine patients in this category displayed hypomethylation in satellite α (centromeric; Figure 1) DNA while all five examined patients who had mutations in *DNMT3B* did not have the pericentromeric DNA hypomethylation extending into the centromere [7]. ICF patients without mutations in *DNMT3B* exhibit the same Sat2 hypomethylation and chromosomal anomalies at 1qh and 16qh in mitogen-stimulated cells seen in patients with *DNMT3B* mutations [8, 13, 14, 48].

## Variability of clinical aspects

### Immunodeficiency

The immunodeficiency displayed by ICF patients, despite the presence of B cells, results in severe recurrent infections of ten seen in early childhood [49–51]. ICF immunodeficiency is variable ranging from agammaglobulinemia to a mild reduction in immune response [10, 52]. In one study, 27 out of 44 patients presented with agammaglobulinemia although they had B cells in the peripheral blood [10]. All but one of the rest hadhypogammaglobulinemia, one with selective I gA, two with I gM, and one with I gG2 subclass deficiency. Normal percentages of T cells were observed in 36 of 38 ICF patients, the expected stimulation of T-cell proliferation in 25 of 28 tested patients, and CD4^+^ cells were decreased in only five of 38 patients.

### Other symptoms

The dysmorphic facial features of ICF are variable, usually mild [50, 53], and frequent [10]. The typical facial features are abroad flatnasal bridge, hypertelorism (very widely spaced eyes), epicanthic folds and low-set ears. Less frequent but still often associated with the syndrome are micrognathia (small jaw) and macroglossia (protrusion or enlargement of the tongue) [6, 28, 53–56]. Failure to thrive and low birth weight are observed in about half of the patients [10]. Mental retardation and neurologic defects have been seeninabout one-third of the patients [10, 50, 53, 57]. Other congenital abnormalities in ICF are highly variable being observed in one or a few patients. These include bipartite nipples [57], skin pigment changes [12, 57, 58], scleral telangiectasias [57], inguinal hernia and cleft palate [10].

### How similar are the Dnmt3b mutant mice to human ICF patients?

Two missense ICF mutations were introduced into both *Dnmt3b* alleles in mice by homologous loxP/cre-driven recombination [17]. Both affect the catalytic domain in the C-terminal portion of the protein [2, 4]. Three ICF-related transgenic mouse lines were generated that were homozygous for one or the other of these *Dnmt3b* mutations or the compound heterozygote.

The missense *Dnmt3b* mutant mice went to term unlike the equivalent *Dnmt3b* homozygous null mice, which died between 13.5 and 16.5 day post coitum [17]. Most of the missense mutant mice died within 1 day of birth although some survived through to adulthood. Those that survived displayed low birth weight and facial anomalies (shorter nose and wider nasalbridge) reminiscent of the ICF syndrome. Although their immune system was abnormal, the identified abnormality was in the T-cell lineage. One or two days after birth, they had reduced amounts of thymocytes, apparently due to apoptosis, and high levels of fragmented nuclei in the thymus. In contrast, the immunodeficiency of ICF patients only in frequently involves decreased levels of T cells [1, 10] but always is characterized by reduced levels of one or more of the types of immunoglobulins. Normal levels of B cells were observed in these mice as is usually the case for ICF patients.

As described above, only ICF Type 2 patients (who do not display *DNMT3B* mutations) exhibit hypomethylation of centromeric satellite DNA (Figure 1). The *Dnmt3b* missense mutant mice displayed hypomethylation of murine minor satellite DNA, which is centromeric, as well as of major satellite DNA, which is juxtacentromeric [17]. No mention was made in this study of chromosomal rearrangements. However, in an earlier report from this group, aneuploidy, polyploidy and numerous breaks and fusions in chromosomes were observed in murine embryonic fibroblasts with double knockout of *Dnmt3b* [59]. This is very different from the chromosomal abnormalities in ICF, which do not include aneuploidy or polyploidy, and are localized almost exclusively to the juxtacentromeric heterochromatin of only afew chromosomes, those with long regions of Sat2 [1, 14] (see below). Therefore, the DNA hypomethylation and chromosomal abnormalities in mice with *Dnmt3b* mutations or derived cell cultures is more extensive (including centromeric as well as juxtacentromeric DNA) than in the *DNMT3B*-mutant ICF syndrome in humans, even when comparing the same *DNMT3B* mutations, and the nature of the immune dysfunction is different. These differences between species in a disease that most prominently affects satellite DNA-rich heterochromatin are not surprising. Mice do not have Sat2- or Sat3-like sequences in their genome, where so much of the ICF DNA hypomethylation is concentrated [60]. Moreover, murine chromosomes are acrocentric, rather than mostly metacentric, as for human chromosomes, including those harbouring most of the Sat2 or Sat3 DNA. At the end of this review, we will discuss our hypothesis that Sat2 hypomethylation plays an indirect causative role in ICF.

## ICF DNA hypomethylation and relationship to cancer

### ICF DNA hypomethylation

Juxtacentromeric satellite DNA in normal somatic tissues is generally highly methylated [61]. The hypomethylation of satellite DNA sequences in ICF cells at 1qh, 9qh, 16qh, and Y qh appears to be an invariant characteristic of ICF as detected by Southern blot analysis with CpG methylation-sensitive restriction endonucleases and by immunocytochemistry with anti-m^5^C antibodies [13, 14, 60]. By bisulfite based genomic sequencing, Sat2 in ICF lymphoblastoid cell lines (LCLs) and fibroblast cell strains was found to have aboutone-third the methylation of analogous controls [62]. The controls had an average of 69% methylation of CpG dinucleotides. By hairpin genomic sequencing, we recently showed that normal somatic tissues have averages of 81, 12 and 7% symmetrically methylated, symmetrically unmethylated and hemimethylated CpG dyads, respectively (C. Shao, M. Lacey, and M. Ehrlich, submitted).

In addition to Sat2 and Sat3, satellite DNA at Yqh is hypomethylated in ICF cells [13, 60]. A nother class of ICF-hypomethylated heterochromatic sequences is in facultative heterochromatin, the inactive X chromosome of females(X_i_) [60, 63, 64]. Hypomethylation of examined CpG islands on the X chromosome has been found in some, but notall, ICF patients on their X_i_ [1, 51, 63]. ICF-related Yqh and X_i_ hypomethylation is unlikely to be of biological significance because no gender-specific differences in symptoms have been reported for this disease, and, for X_i_ genes, the hypomethylation is often inconsistent from patient to patient. Analysis of methylation of imprinted genes in ICF has not revealed any gene region with consistent hypomethylation among examined patients [52]. Therefore, these gene regions too are unlikely to contribute to the ICF phenotype.

By HPLC analysis of DNA digests, we demonstrated that the hypomethylation of the genome in ICF involves only arather small percentage of the 5-methylcytosine residues, namely 7% hypomethylation in brain DNA [14]. W e confirmed that the methylation abnormality of ICF is confined to asmall fraction of the genome by two-dimensional electrophoresis of restriction digests of DNA from four ICF vs. four control LCLs [65]. Only 13 of the approximately one thousand spots displayed consistent ICF-specific differences, and all but one of these was derived from NBL2 and D4Z4t and emrepeats. These repeats are dissimilar from each other and from Sat2 or Sat3 in sequence, G + C content, and chromosomal location (acrocentric, subtelomeric, or juxtacentromeric) despite their common ICF hypomethylation [65–67]. That only alimited amount of consistent DNA demethylation is associated with ICF, and mostly in DNA repeats, should be taken into account in models of how DNMT3B mutation gives rise to the disease.

### Sperm DNA hypomethylation: Similarities with ICF and cancer DNA hypomethylation

Normal human sperm display shypomethylation in juxtacentromeric Sat2 and Sat3, subtelomeric D4Z4 arrays, and acrocentric chromosomal NBL2 sequences as do ICF cells and somatic tissues [13, 61, 62, 65, 66]. Unlike ICF Type 1, but like Type 2 and a very high percentage of cancers, sperm also displays hypomethylation of centromeric Satα sequences [68, 69]. Sat2, D4Z4, and NBL2 exhibit less methylation in purified normal sperm than do ICF cells [14, 67] (unpub.data).

In mice, juxtacentromeric (major satellite) and centromeric (minor satellite) repeats are hypomethylated in both oocytes and sperm [70]. Imprinted genes and high-copy interspersed repeats are differentially methylated in sperm vs. oocyte DNA [71, 72]. Accordingly, there are differences in the prevalence of transcripts for Dnmt3b, Dnmt3a, Dnmt1, Dnmt1s (a Dnmt1 splice variant), and Dnmt3L during the course of gametogenesis in the male vs. the female germline [21].

Given the involvement of DNMT3B in the ICF syndrome and ICF-linked hypomethylation of the above described tandem repeats, it is clear that this enzyme is necessary during development for normal methylation of these sequences in human somatic cells. The low but appreciable levels of methylation in these repeats in ICF somatic cells, which are higher than those of sperm, might be due to either DNMT3A and/or residual DNMT3B activity. The restructuring of chromatin composition during spermatogenesis [73] might inhibit access of satellite DNA and other large tandem repeats to DNMT3B or facilitate access to as yet uncertain DNA demethylation machinery [74, 75] so as to explain the low levels of methylation of these sequences in sperm.

Frequent increases in methylation of some DNA sequences and decreases in methylation of others are seen in a wide variety of cancers [61]. There is often more hypomethylation than hypermethylation of DNA during carcinogenesis, leading to a net decrease in the genomic 5-methylcytosine content [76]. A lthough the exact methylation changes between different cancers of the same type are not the same, there are cancer type-specific differences in the frequency of hypermethylation or hypomethylation of certain genomic sequences. These opposite types of DNA methylation changes appear to be mostly independent of one another, although they may arise because of a similar abnormality leading to long-lasting epigenetic instability in cancers [77]. Evidence of hypermethylation of some DNA sequences in ICF has been sought but has not been found. Therefore, unlike cancers, ICF DNA has exhibited only hypomethylation.

## ICF chromosomal rearrangements and relationship to rearrangements in cancer

### ICF-type chromosomal rearrangements

Diagnosis of ICF usually involves cytogenetic detection of chromosome rearrangements targeted to Sat2-rich 1qh and 16qh [9, 53] (Figure 1). Standard metaphase analysis of mitogen-stimulated lymphocytes from peripheral blood of ICF patients reveals predominantly chromosome breaks, whole-arm deletions, multi-branched chromosomes with upto ten arms of Chr1 and/or Chr16, and, less frequently, translocations, and isochromosomes (usually containing two 1q arms fused in the juxtacentromeric region) [1, 53]. Frequently, there is decondensation in 1qh or 16qh in ICF LCLs or stimulated lymphocytes [14, 78]. This decondensation is probably related to the shift in replication timing of Sat2 from mostly G2/M and very late in S phase and towards more of the Sat2 replicating in S phase, including in intermediate stages of S phase [62].

The decondensation of 1qh and 16qh in ICF lymphoblastoid cells is likely to be critical to the ICF-type rearrangements in these regions. Indirect evidence for more frequent somatic paring of 1qh and 16qh in ICF lymphocytes than in control lymphocytes was reported [79]. In ICF LCLs, multiradials composed of arms of both Chr1 and Chr16 had been shown to be favoured over homologous associations [14]. We had proposed that those multiradials represent unresolved Holliday junctions. In collaboration with David Gisselsson, we analyzed chromosome dynamics at mitosis and the frequency of genomic imbalances in ICF LCLs [80]. Consistent with our model, the results suggest that illegitimate recombination of heterochromatic sequences at interphase due to increased 1qh and 16qh associations in ICF LCLs leads to severe perturbations at mitosis.

DNMT3B co-localizes throughout mitosis with components of the chromosome condensation machinery (hSNF2H, KIF4A, hCAP-C, and hCAPG) in HeL a cells [35]. These proteins were associated with Sat2 and rDNA in interphase as determined by chromatin immunoprecipitation of a control LCL, although the extent of association was not quantitated. In ICF LCL s, one of these proteins, hSNF2H, was tested for its association with DNMT3B and found to still coprecipitate with the mutant DNMT3B [34]. Nonetheless, these proteins and HP1, which is highly concentrated in G2-phase ICF lymphoblasts in an anomalous giant nuclear body containing Sat2 DNA [40], might have a role in the ICF-specific chromosomal abnormalities at 1qh and 16qh.

Because a DNMT inhibitor can give distinctive ICF-like rearrangements in apro-B-cell line, including multiradial chromosomes [29, 30] and can cause decondensation of constitutive heterochromatin [81], it is likely that ICF-linked Sat2 hypomethylation contributes to the ICF-associated 1qh and 16qh abnormalities. Other studies of human lymphocytes and B-cell lines also support a relationship of DNA hypomethylation *per se* and ICF-type chromosomal abnormalities [82, 83] as does the association of juxtacentromeric DNA demethylation and chromosome rearrangements in cancer (see below). Nonetheless, hypomethylation of satellite DNA in constitutive heterochromatin in ICF cells does not suffice to promote rearrangements because the long 9qh region (Figure 1) with its Sat3 hypomethylation in ICF only very infrequently displays rearrangements in mitogen-stimulated lymphocytes from ICF patients [9, 14]. The even longer Yqh region (Figure 1), which also is hypomethylated in ICF cells [13], has not been reported to be susceptible to rearrangement in ICF cells.

Moreover, the cell type and cell growth conditions influence the association of rearrangements with Sat2 hypomethylation. While Sat2-hypomethylated chorionic villus cultures have an increased frequency of these rearrangements, they are only seen in a very low percentage of the metaphases in cultures at low passage numbers [84]. That frequency increases dramatically at higher passages. In ICF cells, the frequency of chromosomal rearrangement sat1qh and 16qh depends on cell growth [1, 9]. *In vitro* mitogen stimulation of lymphocytes greatly increases the development of these aberrant chromosomes independent of its role in inducing cycling. A much higher frequency of juxtacentromeric (pericentromeric) rearrangements of Chr1 and Chr16 per metaphase is seen 72 or 96h after mitogen stimulation of ICF lymphocytes than at 48h, although the frequent abnormal decondensation of 1qh and 16qh can be observed in metaphases at 48 h [12, 53, 85]. The rearrangements observed in mitogen-stimulated ICF lymphocytes and in untreated ICF LCLs may occur *in vivo*, but at a very low rate, as deduced from studies of micronucleus formation in unstimulated bone marrow and lymphocytes from ICF patients [53, 58, 86]. The viability of ICF patients and cell-type specificity of the disease, mostly an immunodeficiency disease, indicates that a generalized breakdown in 1qh and 16qh chromatin stability is not manifest throughout the tissues of ICF patients.

### Relationship of chromosomal rearrangements in ICF to similar rearrangements in cancer

ICF-type chromosome rearrangements have been seen in cancers [87]. Chr1/Chr16 multiradial chromosomes, which are expected to be very short-lived structures [14], and decondensation in Sat2-rich 1qh have been observed in multiple myeloma and hepatocellular carcinomas [88, 89]. In urothelial carcinomas and glioblastomas, there is evidence for an association of DNA hypomethylation with juxtacentromeric chromosomal rearrangements [90, 91]. Unbalanced Chr1 and Chr16 juxtacentromeric rearrangements are over represented in a wide variety of cancers [92]. The unbalanced nature of these rearrangements could foster tumorigenesis by resulting changes in copy number of oncogenes or tumor suppressor genes [61]. Studies of mice with genetically engineered DNA hypomethylation due to hypomorphic mutations in *Dnmt1* provide further evidence that DNA hypomethylation can predispose to chromosome rearrangements [93].

### Cancer incidence in ICF patients

Studies from DNA-hypomethylated mice give evidence of a causal relationship between genomic hypomethylation and cancer, but only certain types of cancer [77, 94]. Although ICF had not been associated with cancer, fewer than 50 patients (mostly children) have been identified. Their usually very short average lifespan would preclude detection of a cancer predisposition that was not very high and did not result in tumors rather quickly. However, recently an ICF patient of 7 years was reported to have Hodgkin lymphoma [95], and previously an unrelated ICF patient was described as having an adrenocortical adenoma [8]. The normal or heightened DNA damage response observed in ICF lymphocytes [96] might help explain why tumors have not been found more frequently in ICF patients compared to patients with shortlife expectancies due to several other rare chromosomal in stability syndromes [97].

## Altered gene expression in ICF lymphoblastoid cells

### Overview of ICF microarray expression analyses

Because ICF patients can have very large decreases in specific serum immunoglobulins despite the usually normal levels of B cells [5, 10], transcriptional dysregulation in B cells or both B and T cells is likely to be the predominant cause of their immunodeficiency. We showed that ICF LCLs have plentiful surface IgM and normal IgM RNA levels [5] despite extremely low levels of serum IgM. Therefore, the immune defect in ICF occurs at a step prior to class switching. It was suggested that peripheral blood-derived B cells and BLCLs in ICF patients may display an altered expression pattern only as result of being less mature than their normal counterparts [98]. However, the absence of detectable IgM in serum from 12 out of 45 patients [10] indicates an intrinsic defect in B cells in ICF patients. Moreover, the differences in the expression patterns of ICF LCLs compared to control LCLs described below argue for more than just a loss of maturity of B cells. For example, we found significant differences in RNA levels in ICF vs. control LCLs for some genes expressed only in mature B cells, others known to be expressed mainly in the T-cell lineage, and yet others with no known or expected relationship to lymphogenesis.

We did two microarray expression experiments on ICF and control LCLs. They involved B-cell lines from ICF patients with known and diverse *DNMT3B* mutations and from controls, including phenotypically normal parents of the patients. In the first study, total RNA (cRNA) from ICF LCLs derived from five different patients and five control LCLs were examined on oligonucleotide arrays for ∼8400 genes (Affyarray HuGene FL, Affymetrix) [5]. The fold change (FC) was determined, namely, the relative RNA signal levels in ICF vs. control or control vs. ICF. About 0.3% of the genes were found to be up- or down-regulated at a significance level (two-sample *t*-test) of *P* < 0.05 and FC > 2.0 or *P* < 0.01 and FC > 1.5. In the second experiment, eight ICF and seven control LCLs, including five and two from the first study, respectively, were analyzed on oligonucleotides arrays representing >33,000 genes (Affyarray U133A and B arrays). In this analysis, ∼1% of the genes showed an FC > 2 (up- or down-regulation) and *P* < 0.01 for ICF-specific differences in RNA levels. Dysregulation involved 120 probesets that were down-regulated and 229 up-regulated.

The summary data for 20 of these genes of interest from the second experiment are shown in Tables I and II. Nine were also found to be dysregulated in the first microarray experiment and eight were similarly up- or down-regulated in two different probe sets in the second experiment. By quantitative real-time RT-PCR (qRT-PCR), we verified that the following RNAs were overexpressed in eight ICF LCLs compared to eight control LCLs: the transcription factor *NR2F2* (*COUP*-*TFII*); *SMARCA2* (*BRM*), encoding a *SNF2* subunit of a chromatin remodelling complex; *PRKCH* and *PTPN13* (*FAP-1*), which regulate apoptosis; *GUCY1B3* and *GUCY1A3*, which encode the two subunits of a soluble guanylate cyclase, and *CD44* and *CKLF*, which are implicated in the later stages of B-cell development. Although the terms up- or down-regulation are used, a caveat is that differences in post-transcriptional processing are sometimes responsible for changes in the steady-state levels of RNA, the parameter monitored in these studies.

**Table I:** Immune system-related genes with significant differences in RNA levels in ICF vs. control lymphoblasts.

				Microarray data^a^					qRT-PCR data^b^	
					
Abbreviated gene name	Ref seq. ID or other ID	Chrom. location	Description	Array expt. no.	Mean for ICF	Mean for controls	FC	*P*-value from *t*-test	FC	*P*-value from *t*-test
IGHG3	M87789	14q32.33	Immunoglobulin heavy constant gamma 3	1	291	>18,600	−60	0.004	NA	
	211647_x_at		Ig rearranged mu-chain gene V-N-D-N-J-region, complete cds	2	62	8765	−100	2 × 10^−8^		
NT5E (CD73)	NM_002526	6q14-q21	5′-Nucleotidase, ecto; hydrolyzes extracellular nucleotides; intracellular signalling, lymphocyte proliferation, activation, and cell cohesion; much greater activity in mature than immature B and T cells	2	377	158	+2.4	0.005	NA	
MAP4K4	NM_145687	2q11.2	Mitogen-activated protein kinase kinase kinase kinase 4; role in T-cell antigen-mediated responses, in cell motility, and in TNF-α/JNK and p53 signalling pathways	2	890	14	+18	0.001	NA	
CASP1	NM_033292	11q22.3	Caspase 1, apoptosis-related cysteine peptidase (interleukin 1, beta, convertase); in B cells, activation of NF-κB and MAPK14	2	961	333	+3.0	0.009	NA	
CD27	NM_001242	12p13	Tumor necrosis factor receptor; family, member 7 (TNFRSF7); T-cell and B-cell proliferation response to antigens; somatically mutated, germinal center, memory, and plasma cells	1	1439	2890	−2.0	0.04	NA	
				2	1552	3081	−2.0	0.03		
ITGB2 (CD18)	NM_000211	21q22.3	Integrin, beta 2 (complement component 3 receptor 3 and 4 subunit); cell surface adhesion receptor involved in B-cell differentiation and homing	2	1046	357	+2.9	0.03	NA	
CD44	NM_000610	11p13	CD44 antigen; attachment to endothelial cells and homing to peripheral lymphoid organs and sites of inflammation; naïve and memory B cells, not on germinal center B cells	2	4651	1438	+3.2	0.005	+3.5	0.01
CD40	NM_001250	20q12-q13.2	Tumor necrosis factor receptor superfamily, member 5; interaction with its T-cell ligand needed for B-cell proliferation, differentiation, isotype switching, humoral memory response; mature B cells	2	1460	487	+3.0	0.01	NA	
CKLF	NM_016326	16q22.1	Chemokine-like factor; chemotaxis; upregulated in activated T cells but not in B cells; very high expression in testes	2	1599	351	+4.6	0.003	+3.3	0.03

a The first [5] and second experiments on ICF vs. control B-cell lines were done similarly on HuGene FL and HG-U133 microarrays, respectively (Affymetrix). The ICF LCLs for Expt. #2 were those in Expt. #1 (only one of the two ICF B stocks) with three additional LCLs, patients 1 and 3 from one study [29] and patient 5 from another [6]. The control LCLs for Expt. #2 were from the mothers and fathers of patient ICF B and C and AG15022 and AG14953 (Coriell Institute). A positive fold change (FC) denotes RNA overexpression in ICF and is the mean signal for ICF divided by that for the controls for that probe set. A negative FC indicates underexpression in ICF and is the negative of the mean signal for controls divided by that for ICF. *P*-values are for two-sample *t*-tests to evaluate the significance of ICF-associated increases or decreases in mean RNA levels relative to the controls. For IGHG3, PRKCH, CD44, and CKLF, another probe set in the microarray gave similar results in Expt. #2 (not shown);

b Real-time quantitative RT-PCR (MyIQ Cycler and iQ SYBR Green Supermix, Bio-Rad) was done on all the ICF LCL samples used for Expt. #2 and 6–12 control LCLs (including additional B-cell lines) that were prepared from random hexanucleotide or oligo(dT)-primed cDNA. Primers were designed for the HG-U133 microarray probe regions and optimal annealing temperatures for PCR were determined by gradient PCR. The data were normalized to those from GAPDH. The fold change is described as for the microarray data. The *P*-values are for the differences in the mean RNA levels using log-transformed data. NA, not assayed.

**Table II:** More genes with significantly altered RNA levels in ICF vs. control lymphoblasts.

				Microarray data^a^					qRT-PCR data^a^	
					
Gene symbol	Ref seq. ID	Chrom. location	Description	Array expt. no.	Mean for ICF	Mean for controls.	FC	*P*-value from *t*-test	FC	*P*-value from *t*-test
PRKCH	NM_006255	14q22-q23	Protein kinase C, eta; calcium-independent and phospholipid-dependent; a PRKCH cleavage product is implicated in apoptosis in pro-B cells but expression in T cells is much higher than in B cells; expression correlated with NO production	1	379	100	+3.8	0.04		
				2	2301	170	+13.6	0.003	+6.5	0.04
GUCY1A3	NM_000856	4q32.1	Guanylate cyclase 1, soluble, alpha 3; heterodimerizes with beta subunit to give an enzyme (sGC) strongly activated by NO	1	233	101	+2.3	0.03		
				2	1193	417	+2.9	7 × 10^−5^	+6.8	4 × 10^−6^
GUCY1B3	NM_000857	4q32.1	Guanylate cyclase 1, soluble, beta 3; heme-containing subunit of sGC; MAPK13 is a downstream effector and HMOX1 is upstream	1	1316	292	+4.5	0.007		
				2	3759	782	+4.8	0.001	+10.6	1 × 10^−5^
HMOX1	NM_002133	22q13.1	Heme oxygenase (decycling) 1; ubiquitious transcriptionally inducible stress protein; degrades heme; generates CO for anti-proliferative effects on T cells	1	577	2353	−4.1	0.03		
				2	864	1559	−1.8	0.009	NA	
MAPK13	NM_002754	6p21.31	Mitogen-activated protein kinase 13; stress activated kinase; activated by PRKCH during regulation of keratinocyte differentiation; downstream in the sGC pathway; is pro-apoptotic; implicated in antigen-meidated T-cell responses	2	388	104	+3.7	0.006	NA	
PTPN13	NM_080683	4q21.3	Protein tyrosine phosphatase, non-receptor type 13 (APO-1/CD95 (Fas)-associated phosphatase); both anti- and pro-apoptic effects; IκBα is a substrate; high expression in placenta and testis	1	271	41	+2.7	0.02		
				2	623	53	+12	1 × 10^−7^	+5.0	0.02
BCL2L10	NM_020396	15q21.2	BCL2-like 10 (apoptosis facilitator); both anti- and pro-apototic	2	560	184	+3.0	0.002	NA	
CNN3	NM_001839	1p22-p21	Calponin 3, acidic; actin-binding protein; low expression in leukocytes	1	407	80	+4.1	0.008	NA	
				2	1046	357	+2.9	0.03		
SLC1A1	NM_004170	9p24	Solute carrier family 1 (neuronal/epithelial high affinity glutamate transporter)	1	137	237	−1.7	0.001	NA	
				2	716	1493	−2.1	0.007	NA	
NR2F2	NM_021005	15q26.2	Nuclear receptor subfamily 2, group F, member 2; COUP-TFII; negative post transcriptional regulator of MYOD1	2	824	91	+9.0	0.003	+2.9	0.005
SMARCA2	NM_003070	9p24.3	SWI/SNF related, matrix associated, subfamily a, member 2; BRM; chromatin remodelling	2	2814	1250	+2.1	0.002	+3.3	0.004

a See the footnotes to Table I. Two probe sets each for GUCY1B3, CNN3, SMARCA2, and BCL2L10 in microarray Expt. #2 gave similar results.

Twelve genes with significant differences from the microarray data between ICF and control LCLs were then tested by RT-PCR. Only one of these did not exhibit RT-PCR results concordant with the micro-array results (data not shown). In addition, one gene, *RGS1*, which displayed ICF-specific changes in its RNA levels only in the first microarray experiment, did not show significant differences in RNA levels in ICF vs. control LCLs by qRT-PCR (data not shown). Some other genes also displayed significant differences in RNA levels between ICF and control LCLs only in the first experiment [5]. However, the microarray for the first experiment was smaller, the gene annotation was much less thorough, and the probe sets were different from those in the second experiment (usually 20 oligonucleotides per set in the first experiment and 11 in the second). These factors and the absence of some of the probe sets or the apparently poorer hybridization of others can explain why some of the genes that appeared to be significantly dysregulated in ICF LCLs compared to controlLCLs in the first array experiment were not seen as dysregulated in the second.

In view of the extreme scarcity of ICF patients and their median age of 8 years at death [10], we examined Epstein Barr virus-transformed B-cell lines rather than lymphocytes. The activation associated with this transformation might hide some *in vivo* defects in B-cell activation in ICF patients and only asubset of B cells will be transformed. Nonetheless, we found much consistency in LCLs from eight unrelated patients and new insights into transcriptional regulation of the immune system.

### Genes with lymphocyte functions that had altered RNA levels in ICF LCLs

The most dramatic difference in RNA levels in ICF vs. control LCLs was seen for IGHG3 (Table I). This was expected based upon results given results from the patient sera and surface immunoglobulin expression [5]. The second largest difference in RNA levels in disease compared to control LCLs in Table I or II was observed for MAP4K4, which has been implicated in antigen-mediated T-cell activation [99] but not in B-cell function. This ubiquitously expressed kinase participates in the JNK/TNF-α and p53 signalling pathways and can be controlled at the level of transcription [100–102]. The maximum microarray signal for MAP4K4 in the control LCLs in the second microarray experiment was 46 while, with the exception of one LCL (ICF K), the ICF LCLs had much higher signals (371, 2893, 295, 291, 1856, 348 and 1060). MAP4K4 seems to have abroad role in fostering cell migration [103]. A nother gene whose RNA was upregulated in ICF vs. control LCLs was *NT5E*, which was found to be expressed mostly in B cells, rather than T cells, and usually only after isotype switching [104].

CD27, which plays a key role in T-cell memory and in the stimulation of B cells to produce immunoglobulins [105], had lower mRNA levels in ICF LCLs relative to control LCLs (Table I). This is consistent with ICF immune dysfunction. The ability of peripheral blood-derived B cells (CD19^+^) to express cell-surface CD27 after stimulation [98] makes the down-regulation of its RNA in ICF lymphoblastoid cells unlikely to be causally involved in ICF immunodeficiency. While CD40, CD44, CKLF and ITGB2 (CD18) mRNAs were significantly upregulated in ICF vs. control LCLs, their positive functions in lymphogenesis [106–112] make them unlikely candidates for active players in the immunodeficiency of ICF patients. Some gene candidates for interfering with later stages of B-cell differentiation or activation did not display significant differences in RNA levels in ICF vs. control LCLs in the microarray analysis, namely, *BTK*, *PRDM1*, *PAX5*, *IRF4*, *BCL6*, *XBP1*, *BACH2* and *MAPBPIP*.

### Cell death or growth arrest genes that had altered RNA levels in ICFLCLs

Genes involved in cell death or arrest of the cell cycle may be important contributors to the immune dysfunction of ICF patients. *CASP1*, *BCL2L10*, *PTPN13*, *HMOX1*, *MAPK13* and *PRKCH*, which displayed ICF-specific differences in their RNA levels, might be involved in abnormal regulation of apoptosis or cell cycle arrest in lymphoid cells in ICF patients (Tables I and II). However, major decreases in numbers of lymphocytes are not usually found to be associated with ICF. Low levels of T cells are present in only half of ICF patients and the levels of B cells are even less likely to be lower than normal in ICF patients and are never undetectable [1, 9]. Nonetheless, too much cell death induced by B-cell activation just in the later stages of B-cell development could lead to the loss of plasma cells and decreased serum immunoglobulin without compromising total levels of B cells.

CASP1 (Table I) is a cytokine involved in a variety of inflammatory processes, including the proteolytic maturation of the inactive precursor of the inflammatory cytokines interleukin-1 (IL1) and IL18 [113]. It is pro-apoptotic in various cell types and may be associated with IgA deficiency and increased apoptosis in B cells [114]. Therefore, the observed increased levels of CASP1 RNA in ICF vs. control LCLs are consistent with the heightened susceptibility of ICF lymphoid cells to apoptosis [80, 86, 115, 116]. Over-expression of CASP1 RNA in ICF cells could also perturb NF-κB signalling pathway simpacting expression of other genes (see below). Underexpression of RNA for the anti-apoptotic HMOX1 stress protein and overexpression of MAPK13 and PRKCH RNA in ICF vs. control LCLs (Table II) might also contribute to pro-apoptotic tendencies of ICF lymphoid cells.

There was significantly more BCL2L10 RNA in ICF LCLs compared to control LCLs (Table II). This widely expressed member of the BCL2 family has a polypeptide structure compatible with both pro-and anti-apoptotic effects depending on the cell type and conditions [117]. It appears to be a negative regulator of cell death in humanglioma cells provoking them to exit from the cell cycle [118]. While ICF LCLs were hypersensitive to γ gradiation compared to controls, we demonstrated that this was mostly due to irreversible growth inhibition, secondarily to non-apoptotic cell death, and thirdly to apoptosis [96]. All three of these types of responses to irradiation were significantly more frequent for ICF cells than for control cells despite the functional cell cycle checkpoints in ICF cells.

Given that the main anti-proliferative response of ICF LCLs to γ radiation was not apoptosis, it is noteworthy that *PTPN13*, a gene with anti-apoptotic effects was overexpressed in mRNA from ICF vs. control LCLs (Table II). This protein tyrosine phosphatase interacts with diverse proteins including the cell death FAS protein [119]. The interaction with FAS decreases the export of FAS to the cell surface and there by opposes the FAS pro-apoptotic activity. PTPN13 may suppress pro-apoptotic signalling also through another interactive partner, a neurotrophin receptor [120]. However, as for BCL2L10, an opposite pro-apoptotic role for PTPN13 has also been reported [121], in this case, involving dephosphorylation of the insulin receptor substrate-1. Northern blotting revealed very strong signals for PTPN13 RNA inplacenta and testes (both tissues with considerable DNA hypomethylation [61, 122]), as well as for kidney; moderate signals in lung and ovary; weak signals in heart, brain and pancreas; and almost nodetectable signal in leukocytes [123]. With the much more sensitive RTPCR assay, PTPN13 RNA was detected in T cells and was found to be decreased upon IL-2 activation although it was more abundantin CD45RO^+^ memory T cells than in CD45RA + naive T cells [124].

### Overlapping upregulation of RNA for kinases and NO or CO signal pathway members

The microarray results suggest that overlapping signal transduction pathways may be critical for the immune dysfunction of the ICF syndrome (Tables I and II). One of these involves the a forementioned CASP1 and PTPN13, whose RNAs were upregulated in ICF vs. control LCLs. In addition to its catalytic function, CASP1 has anon-catalytic role, as activator in B cells of NF-κB and p38MAPK, the MAPK family that includes MAPK13 [125]. An increase in NF-κB signalling is also predicted from increased PTPN13 RNA, because the corresponding protein phosphatase has as one of its substrates IκBα, which inhibits NF-κB [126]. In addition, NF-κB upregulates *PTPN13* transcription [119] and plays a critical role in lymphocyte development and function [127, 128].

Another signalling pathway with ICF-specific differences in RNA for several of its members is the carbon monoxide (CO) pathway (Table II). HMOX1, which displayed ICF-specific decreases in RNA levels, catabolizes heme and there by releases gaseous CO, which is responsible for its anti-apoptotic effects [129]. This pathway also involves downstream activation of NF-κB, which, in turn, by promoter interactions, activates transcription from a subset of NF-κB-dependent anti-apoptotic genes [129–131]. Among the diverse effects of CO signalling, it seems to be a modulator of autoimmunity [129].

Another link to the NF-κB signal pathways among the ICF-overexpressed RNAs involves the above mentioned protein kinase C family member, PRKCH. This calcium-independent, serine-and threonine-specific enzyme is activated by diacylglycerol to phosphorylate a wide range of cellular proteins and there by influence many aspects of physiology [132–135]. Un likely most protein kinase C isoforms, transcription of *PRKCH* is highly tissue specific. Its expression primarily, but not exclusively, in epithelial tissues is probably due to an enhancer, a silencer and trans-acting factors [136]. In skin, PRKCH is associated with terminal differentiation of keratinocytes. Genetic polymorphisms in *PRKCH* are implicated in increasing the risk of rheumatoid arthritis and cerebral infarction [135, 137]. Overexpression of PRKCH may play a role in tumor progression through downstream ERK and ELK effectors [132]. PRKCH RNA was overexpressed 4- to 14-fold in ICF vs. control LCLs as seen in both microarray experiments and qRTPCR (Table II). It is implicated as a pro-apoptotic protein in early B-cell development [138]. PRKCH RNA was reported to be induced upon lymphocyte activation but was present at much lower levels in B cells than in T cells, whether resting or activated [137]. A mong the processes subject to its regulation, PRKCH helps control the activation of NF-κB upon lipopolysaccharide induction of primary rat astrocytes [133]. PRKCH activity can result in the production of nitric oxide (NO), another important signalling gas, via the inducible nitric oxide synthetase gene (*iNOS*) [134].

The widespread signalling molecule NO is, in turn, related to GUCY1A3 and GUCY1B3, whose RNAs were upregulated in ICF compared to control LCLs as determined in both microarrays and by qRT-PCR (Table II). GUCY1A3 and GUCY1B3 are the two subunits of the soluble guanylate cyclase (sGC), a heme protein that is a major sensor for NO, thereby regulating diverse physiological processes, [129]. Proliferation of Thelper (Th) 1 cells is controlled by NO levels [139]. Although *GUCY1A3* and *GUCY1B3* are very close together on 4q32.1, they may not be regulated in a fully coordinate manner [140], as is consistent with our data (Table II). Their heterodimeric products GC is strongly stimulated by NO and weakly stimulated by CO. The NO and CO pathways intersect in various ways at the protein level, including that decreased HOX1 in ICF vs. control LCLs could lead to less HOX1-mediated degradation of the hemeprosthetic group necessary for the GUCY1B3 subunit of sGC [141]. Another pathway intersection of the genesin Tables I and II is that MAPK13 has a prominent role as a downstream enzyme in the sGC signalling pathway [129]. ICF-related overexpression of RNAs for MAPK13 and the two sGC subunits is consistent with their interrelated functioning in cell growth arrest.

Some of the genes in Tables I and II that exhibited ICF-related changes in RNA levels in the B lymphoblastoid cells are much more closely associated with T cells than with B cells. This might reflect coordinate dysregulation in the B-cell and T-cell lineages. For those genes, it might be that only dysregulation in T cells is relevant to the disease or that the role of these genes in B cells is insufficiently appreciated. Alternatively, these changes might contribute to the pathology of ICF because of inappropriate expression of T-cell specific genes in the B-cell lineage.

### Dysregulation of genes that may help explain non-immune symptoms of ICF

While the significantly altered RNA levels for some proteins in ICF vs. control LCLs, such as the CNN actin-binding protein, might have no biological consequences, others may be a factor in the nonimmune symptoms of ICF. Although we examined RNA only in BLCLs, abnormal RNA levels in the lymphoid cell lineage might be found in other lineages too and altered regulation in lymphocytes can sometimes mirror more physiologically important dysregulation in a very different tissue [142]. Overexpression of PTPN13 RNA in ICF LCLs (Table II) might pertain to neurological findings in ICF patients because high levels of PTPN13 in fetal brain and its ability to bind to the neurotrophin receptor implicate this protein in controlling neuronal cell death [120, 143]. One of the neurological abnormalities in ICF is seizures, which was reported in three of 45 patients [10]. SLC1A1, a neuronal protein involved in transporting glutamate that is protective against seizures and neuronal death [144], was significantly underexpressed at the RNA level in ICF LCLs relative to control LCLs (Table II). In addition, the observed BCL2L10 RNA dysregulation may be involved in ICF-related neurological disturbances because BCL2L10 mRNA increases appreciably from a very low level during *in vitro* differentiation of rat astrocytes [145]. Moreover, GUCY1B3 and iNOS are associated with each other in certain areas of the hippocampus in mice [146] and CASP1 overexpression has been linked to cognitive impairment with aging [147].

Upregulation of PRKCH (Table II) may explain a perplexing finding reported in one study of two unrelated ICF patients, both of whom displayed bipartite nipples [57]. PRKCH is implicated in upregulation of many epithelial tissues, including the mammary gland in rats [148]. These associations might account for the occasional specific finding of bipartite nipples in ICF patients.

ICF-associated upregulation of RNA for the chromatin remodelling protein SMARCA2 might impact 1qh and 16qh decondensation. SMARCA2 (BRM) is an important modulator of chromatin assembly [149]. It could also have disparated own stream effects through its impact on transcriptional regulation, DNA repair and homologous recombination.

### Lack of detectable methylation changes in the promoter regions of examined ICF-upregulated genes

**Figure 2:**
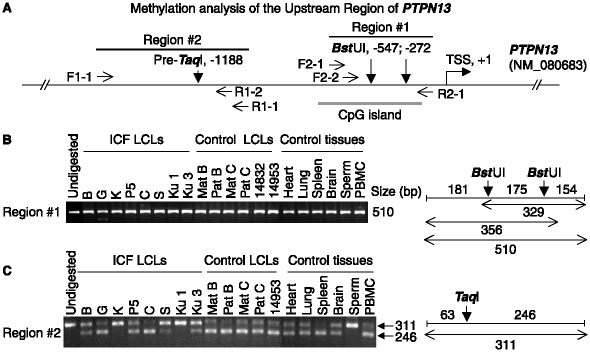
Analysis of DNA methylation upstream of *PTPN13*. Representative COBRA analysis for DNA methylation of a gene that had RNA upregulated in ICF vs. control LCLs. DNA samples had been modified with bisulfite and amplified by PCR with primers at the indicated positions as previously described [5]. The PCR products could be cleaved by TaqI or BstUI only if they had been methylated at the CpG dinucleotide in the indicated sites in genomic DNA; the pre-TaqI site, CCGA, would be converted to aTaqI site, TCGA, upon bisulfite treatment and PCR if it was Cm5 CGA in genomic DNA. (A) Diagram of the 5′*PTPN* region showing the transcription start site (TSS) [123] at Chr4 87,734,909 (hg18, UCSC database), the 5′ CpG island (−701 to −150), and PCR primers; positions are given relative to the TSS. (B) and (C), electrophoresis gels stained with ethidium bromide and visualized for fluorescent bands from the PCR products (−628 to −119 and −1250 to −940) digested with *Bst* UI or *Taq* I. PBMC, peripheral blood mononuclear cells; ICF LCLs are described in the legend to Table I, with the addition of another control LCL (AG14832, Coriell Institute). Sizes are given in bp for the expected and obtained restriction products.

We recently examined methylation of five genes with qRT-PCR-confirmed ICF-associated upregulation of their RNA, namely, *GUCY1A3*, *PTPN13*, *NR2F2*, *SMARCA2* and *CKLF* (Tables I and II). Their 5′ gene region (for *GUCY1A3*) or upstream regions (for the other genes) were assayed for methylation in ICF and control LCLs as well as in several normal tissues by combined bisulfite restriction analysis (COBRA). COBRA allows quantitation of DNA methylation levels at restriction endonuclease sites in a given DNA sequence that is amplified by PCR [150]. Genes with up-regulation of RNA were chosen because of the frequent association of DNA hypomethylation in promoters with transcription [151]. By COBRA, we saw no consistent ICF-specific differences in DNA methylation in the examined regions despite their ICF-related increases in the levels of the corresponding mRNAs (Figure 2 and data not shown). This was similar to our previous finding for *GUCY1B3* in ICF cells [5]. Almost all of the immediate upstream regions were constitutively unmethylated. There were various amounts of partial methylation in their further upstream sequences but no correlations between methylation and RNA levels amongindividual LCLs. Therefore, the differential mRNA levels for these genes in ICF vs. control LCLs could not be explained by differences in methylation in or near their promoters. A caveat in this analysis is that only the top gene in an ICF-activated transcription pathway might have ICF-specific promoter hypomethylation.

## Hypothesis: The relationship of DNMT3B mutations to the ICF phenotype

Methylation analysis of various genes [51, 52, 63, 64] (Figure 2), and whole-genome restriction analysis [65] have revealed consistent hypomethylation only in tandem DNA repeats (including satellite DNA) and X-linked sequences in X_i_. However, unconventional genes, whose broad biological influence has been appreciated only recently (especially micro RNA and anti-sense RNA genes) [152], were not specifically examined. While a critical gene that is hypomethylated specifically in ICF cells may have been missed, we favour a different explanation for the connection of selective DNA hypomethylation to the ICF syndrome. We propose that the pathogenicity of *DNMT3B* mutations in ICF patients is due to the hypomethylation of constitutive heterochromatin. This same explanation can be applied to patients with ICF type 2, who have no detected DNMT3B mutations but do exhibit the characteristic hypomethylation of juxtacentromeric satellite DNA [7]. Because no gender bias has been reported for ICF, our proposal for the involvement of satellite DNA is limited to the long juxtacentromeric heterochromatin butnot Yqh (Figure 1) nor the facultative heterochromatin of X_i_. We favour the involvement of Sat2-containing 1qh and 16qh over Sat3-containing 9qh (Figure 1) because of the more frequent cytogenetic abnormalities in the former regions.

Evidence is mounting that constitutive heterochromatin is biologically important and not just an inert filler in the genome, as many previously thought. In fission yeast and drosophila, transcription of non-coding RNA is important for the establishment of constitutive heterochromatin [153–155]; these organisms have little or no methylation of their DNA [16]. Some, but not all, of various tested normal or cancer samples andhalf of ICF LCLs that we analyzed for Sat2 RNA were positive by RT-PCR (which included controls for DNA contamination) and by assays for RNA polymerase engagement [156]. However, these signals were very low and we did not see the large increase in Sat2 transcripts upon heat shock [156] that is found for 9qh Sat3 transcripts [157].

Besides constitutive heterochromatin yielding non-coding transcripts that might affect expression of protein-coding genes, its intranuclear location may help organize chromatin throughout the nucleus so as to modulategene expression in *trans* [85]. Evidence for this phenomenon has been reported in the lymphoid lineage [158, 159]. The intranuclear distribution of centromeres in lymphoid cells is distinct for the cell type and stage of differentiation and involves genes associated with lymphogenesis [160]. The importance of the spatial location of chromatin in the nucleus is illustrated by the finding that much gene expression occurs in transcription factories that are specific for different functional groups of genes [161]. Hypomethylation of 1qh, 9qh, and 16qh satellite DNA might influence the distribution of these heterochromatic regions in the nucleus during certain stages in lymphogenesis or lymphocyte activation and there by affect expression from genes on other chromosomes.

Recently, one dramatic change in positioning of hypomethylated constitutive heterochromatin specifically in ICF lymphoblasts and lymphocytes has been described. It is the formation of agiant promyelocytic leukemia (PML) type nuclear body that correlated with under condensed 1qh or 16qh, but not 9qh, in a large percentage of ICF G2 nuclei [39, 40]. All three isoforms of HP1 as well as SP100, SUMO-1, transcription factors CBP and DAXX, the DNA helicase BLM, and the SWI-SNF remodelling protein ATRX co-localize in this single nuclear body. M uch smaller PML-type bodies containing these proteins are observed in G2-phase nuclei of normal cells but the association of 1qh Sat2 DNA with these normal bodies is less frequent than for the giant PML nuclear body in ICF lymphocytes and LCLs. This abnormal concentration of satellite DNA heterochromatin and nuclear proteins in ICF G2-stage lymphoid cells has been proposed to be linked to undercondensation and chromosomal abnormalities at 1qh and 16qh [40]. However, it might also reflect an abnormal distribution of chromatin proteins in interphase that could influence expression of genes else where in the genome.

There are more and more examples of transcription control proteins that bind selectively to constitutive heterochromatin [162–168]. Furthermore, there is evidence that the binding of at least some of these transcription factors to the highly repetitive DNA of constitutive heterochromatin sequesters these proteins in a reversible manner so as to modulate expression of sets of genes [5, 168–172]. Methylation of satellite DNA can dramatically alter binding of certain transcription control proteins to DNA, in general [173] or constitutive heterochromatin [174], in particular. Therefore, pathogenic dysregulation of a subset of genes in ICF might be due to altered transcription factor binding to satellite DNA in response to its disease-related hypomethylation. This would be a new type of DNA methylation control of gene expression in *trans* mediated by chromatin acting as a dynamic reservoir for storage and possibly delivery of transcription modulatory proteins, which in the case of ICF, might explain the life-threatening immunodeficiency.
